# *Nakaseomyces glabratus* drug resistance genes expression in vulvovaginal candidiasis: a systematic review

**DOI:** 10.22034/cmm.2024.345248.1573

**Published:** 2024-02-01

**Authors:** Fatemeh Zahra Ranjbar Golafshani, Firoozeh Kermani, Soheila Abbaszadeh Godarzi, Mojtaba Taghizadeh Armaki, Saeid Mahdavi Omran

**Affiliations:** 1 Department of Parasitology and Medical Mycology, School of Medicine, Babol University of Medical Sciences, Babol, Iran; 2 Infectious Diseases and Tropical Medicine Research Center, Health Research Institute, Babol University of Medical Sciences, Babol, Iran; 3 Department of Obstetrics and Gynecology, School of Medicine, Babol University of Medical Sciences, Babol, Iran

**Keywords:** Antifungal drug resistance, *Candida glabrata*, *Nakaseomyces glabratus*, Systematic Review, Vulvovaginal candidiasis

## Abstract

**Background and Purpose:**

Antifungal resistance in *Nakaseomyces glabratus* presents a notable obstacle in the management of vulvovaginitis. Comprehension of drug-resistance gene expression is fundamental in the development of efficacious treatment strategies. This systematic review endeavored to ascertain the existing knowledge regarding the expression of drug resistance genes in *N. glabratus* associated with vulvovaginal candidiasis by the amalgamation of published research findings.

**Materials and Methods:**

Under the PRISMA guidelines, a systematic review was conducted from January 2000 to December 2024, utilizing the articles from "Web of Sciences", "PubMed", and "Scopus". The search incorporated the terms *C. glabrata* complex (*C. glabrata* sensu stricto, *C. nivariensis*, *C. bracarensis*) in conjunction with drug resistance gene expression in *N. glabratus* and closely related species with vulvovaginal candidiasis. The review was restricted to publications in English. The data extraction process employed EndNote software (version 21.4), and a meticulous selection process was undertaken to identify relevant studies.

**Results:**

Three eligible studies reported an increase in the expression of the *CDR1* gene in fluconazole-resistant *N. glabratus*, suggesting the presence of efflux mechanisms that reduce drug accumulation. Another study found enhanced *CDR1* expression in fluconazole-resistant *C. nivariensis*, indicating the existence of similar resistance mechanisms. The observed variations in gene expression profiles between *N. glabratus* and *C. nivariensis* underscore the presence of diverse resistance mechanisms.

**Conclusion:**

Review of the previous studies showed that the *CDR1* gene was the most important resistance gene and that this resistance is more evident in *C. nivarensis*, compared to *N. glabratus*.

## Introduction

In recent years, the emergence of antifungal drug resistance in *Nakaseomyces glabratus* has posed a significant challenge in the management of vulvovaginitis candidiasis [ 1 ]. In 2003, Krutzman classified *C. glabrata* in the class *Nakaseomyces* [ 2 ]. This fungus, a haploid yeast closely related to Saccharomyces cerevisiae, is known to cause yeast infections in humans. *Nakaseomyces glabratus* complex consists of three species known to cause human diseases, namely *C. glabrata* sensu stricto, *C. nivariensis*, and *C. bracarensis* [ 3 ]. *Nakaseomyces glabratus*, as a fungal pathogen, exhibits notable resistance to antifungal drugs, which results in considerable difficulties in treating patients with this disease [ 4 ].

It is an opportunistic fungal pathogen responsible for superficial mucosal infections and life-threatening bloodstream infections in immunocompromised individuals. *Nakaseomyces glabratus* is associated with conditions, such as candidemia, invasive candidiasis, and candiduria [ 5 ]. In addition, *N. glabratus* is the second most common cause of vulvovaginal candidiasis, particularly in recurrent vulvovaginal candidiasis [ 6 ].

Recently, there have been reports of an increasing prevalence of *N. glabratus* as a causative agent of fungal infections among patients in various countries, such as Iran [ 6 - 8 ]. Genetic analyses have revealed that mutations in the *PDR1* and *ERG11* genes significantly contribute to antifungal resistance. However, investigations of candidemia samples in Iran have reported a comparatively low incidence of these mutations, which may be attributable to genetic diversity and variations in treatment protocols [ 5 ,  9 ].

Recent studies have demonstrated upregulation in the expression of resistance genes, such as *CDR1*, *ERG11*, and *MDR1* in *N. glabratus*. These genes are associated with drug efflux mechanisms and can lead to intracellular drug accumulation. Understanding these mechanisms can aid in investigating the precise mechanisms of resistance in these fungi, thereby facilitating the development of novel therapeutic approaches.

This study aimed to perform a systematic review of antifungal resistance genes associated with the *N. glabratus* complex and assess the current state of antifungal resistance gene expression in vulvovaginal candidiasis among the species of this complex.

## Materials and Methods

### Ethical considerations

The study was approved by Babol University of Medical Sciences and was conducted in compliance with the ethical guidelines with the code IR.MUBABOL.HRI.REC.1402.284.

### Search strategy

The present systematic review adhered to international standards and followed the PRISMA checklist. Articles were retrieved from reputable databases, namely "Web of Sciences", "PubMed", and "Scopus" with keywords, such as "gene resistance" OR "efflux pump gene" OR "*ERG* gene" OR "*CDR* gene" OR "*PDR* gene" OR "*MDR* gene" OR "*FSK* gene" OR "gene expression" OR "*YPS1*" OR "*AWP3*" OR "*EPA1*" OR "*ERG11*" OR "*CDR1*" OR "*CDR2*" OR "*ERG6*" OR "*TAC1*" OR "*PDH1*" OR "*SNQ2*" OR "*FAA1*" OR "echinocandin resistance gene" OR "drug resistance gene" OR "azole resistance gene" AND "vulvovaginitis" OR "vulvovaginal candidiasis" AND "*Torulopsis glabratus*" OR "*Candida glabrata*" OR "*C. glabrata*" OR "*Nakaseomyces glabratus*" OR "*N. glabratus*" OR "*C. nivariensis*" OR "*C. bracarensis*" OR "*Candida nivariensis*" OR "*Candida bracarensis*". The review included studies conducted from 2000 to 2024, covering the past 24 years to encompass significant global changes.

### Study selection

Articles eligible for inclusion were those that investigated the expression of drug resistance genes in *N. glabratus* associated with vulvovaginitis. Articles were selected through a rigorous selection process, which included the evaluation of samples, sample sizes, non-response rates, and measurement instruments utilized. Furthermore, articles were compared to ensure that confounding variables and other influencing factors were thoroughly examined. Moreover, articles that were not directly related to the research topic were excluded; only articles evaluating antifungal resistance genes in *N. glabratus* isolated from vulvovaginal candidiasis were included in the review process.

### Data extraction and analysis

To systematically handle the studies, all initially searched articles were imported to the EndNote software (version 21.4, Clarivate Analytics, USA). Initially, duplicate documents were removed. The screening process of articles involved the examination of the titles and abstracts of the studies. In this manner, irrelevant articles were eliminated. Subsequently, the full texts of the remaining articles were reviewed to ensure adherence to the inclusion and exclusion criteria.

**Figure 1:**
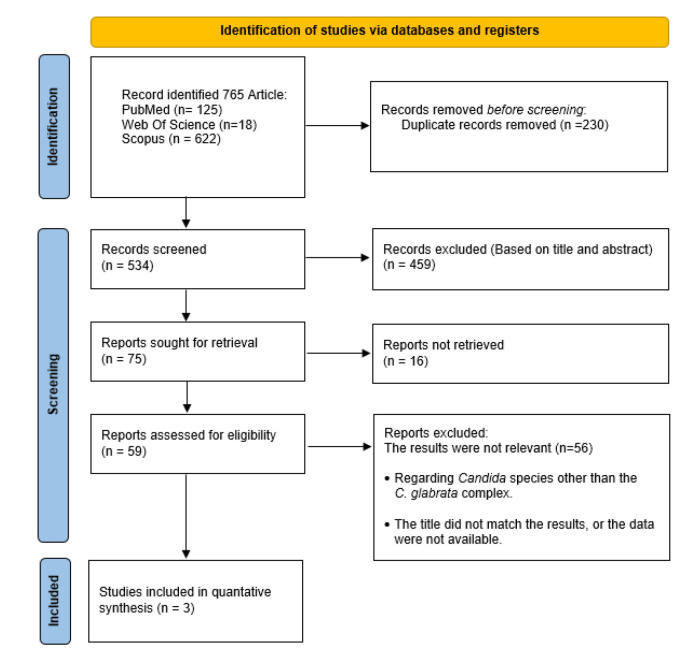
PRISMA 2020 flow diagram for a systematic review of *Nakaseomyces glabrata* drug resistance genes expression in vulvovaginitis.

This process was conducted by two individuals independently. Any discrepancies between these individuals were resolved by a third person, who facilitated the final decision-making process. Two authors extracted the data independently, including names of authors, year of publication, number of isolates, species, and expression of resistant genes (Figure 1).

## Results

A comprehensive search of the databases yielded a total of 765 records. After evaluation of the titles, abstracts, and full text of the eligible articles, 762 articles that were not relevant to the objectives of the study were excluded. Subsequently, the EndNote software was employed and a comprehensive review of the full texts of the remaining studies was conducted. Finally, three articles were selected for inclusion in the study, which successfully met all the specified inclusion criteria (Figure 1).

All three studies observed an increase in the expression of genes associated with antifungal resistance in *N. glabratus* isolated from vulvovaginal candidiasis patients (Table 1). The results indicated the existence of only a significantly limited number of studies on resistance genes in *N. glabratus* isolated from vulvovaginal candidiasis patients, highlighting the importance of addressing drug resistance.

**Table 1:** Resistant genes and virulent genes mRNA expression of *Candida glabrata* and *Candida nivariensis*.

References	Country	Number of isolates	Species	Strain	Expression of resistant genes			
					*ERG11*	*CDR1*	*CDR2*	MDR1
Gygax et al. (2008) [ 10 ]	USA	2	*C. glabrata*	-	NA	≥3-fold increase	NA	≥2-fold increase
Lotfali et al. (2022) [ 4 ]	Iran	2	*C. glabrata*	-	0.366	NA	NA	NA
Shia et al. (2019) [ 11 ]	China	9	*C. nivariensis*	8236	3.91	12.76	12.29	NA
				8283	0.28	2.59	–0.02	
				8472	4.14	15.86	14.42	
				8544	4.28	10.27	8.23	
				8568	3.37	11.61	11.31	
				D164	17.78	19.61	16.68	
				D229	3.06	12.04	10.99	
				E156	1.31	9.96	8.70	
				E384	2.25	12.72	11.96	
				E500	4.90	12.80	12.12	
				F187	1.64	10.85	10.13	
			*C. glabrata*	8581	0.27	3.06	0.12	NA
				9476	0.87	2.70	–0.45	
				S078	1.85	2.61	–0.61	
				S092	–0.48	2.14	–0.45	
				S103	0.05	2.44	1.35	
				S118	0.54	2.36	0.16	
				S148	17.10	16.62	15.99	
				S150	7.69	8.72	7.48	
				S151	2.36	1.91	0.39	
				S163	0.72	2.30	0.82	
				S415	0.00	2.83	–0.19	

* NA: not applicable

Lotfali et al. [ 4 ] examined point mutations in the *ERG11* gene. According to the results of the quantitative reverse transcription polymerase chain reaction, they found that the mRNA expression level of *ERG11* was significantly upregulated in two resistant *N. glabratus* isolates, compared to the reference strains, with observed fold changes ranging from 1.29 to 3.66.

Gygax et al. [ 10 ] reported a significant elevation in *CDR1* and *MDR1* gene expression in fluconazole-resistant *N. glabratus*. These genes are connected to drug efflux mechanisms that can decrease intracellular drug accumulation.

Shi et al. [ 11 ] investigated the overexpression of *ERG11*, *CDR1*, and *CDR2* in fluconazole-resistant *C. nivariensis* relative to *N. glabratus*, revealing a resistance mechanism linked to a comparable efflux pump: *CDR1* (11.9 vs. 4.34), *CDR2* (10.62 vs. 2.24), and *ERG11* (4.27 vs. 2.82). This finding underscores the necessity of evaluation of efflux pump gene expression across different species within the *N. glabratus* collection (Table 1).

## Discussion

Azole antifungal agents, particularly fluconazole and clotrimazole, play a significant role in clinical practice for the management of vulvovaginal candidiasis. *Nakaseomyces glabratus* is a fungal pathogen that is increasingly recognized as a causative agent of vaginitis [ 12 ]. It ranks as the second most common cause of non-*albicans Candida* vaginitis after *C. albicans*. Furthermore, *N. glabratus* exhibits higher resistance to antifungal drugs, compared to *C. albicans*, leading to significant treatment challenges [ 10 ,  13 ].

*Nakaseomyces glabratus* exhibits multiple resistance mechanisms to fluconazole, which include variations in gene regulation, genetic mutations, and cross-resistance among azole derivatives. Reports have shown that there are variations in the virulence factors and antifungal resistance among different species within the *N. glabratus* complex. For example, it has been reported that *N. glabratus* sensu stricto is more susceptible to fluconazole, itraconazole, and voriconazole, compared to *C. nivariensis* [ 14 ].

Catheter-associated candidemia cases caused by fluconazole-resistant *C. nivariensis* have been documented by Fujita et al. (2007), emphasizing the need for alternative treatments. In blood culture susceptibility tests, echinocandins (caspofungin and micafungin) and flucytosine were found to be the most effective treatments in these cases due to their high sensitivity [ 15 ]. Shi et al. [ 11 ] observed that *C. nivariensis* strains had higher minimum inhibitory concentrations geometric means of caspofungin, fluconazole, itraconazole, and amphotericin B, compared to *C. albicans*. Furthermore, conventional antifungals showed a low cure rate in patients with vulvovaginal candidiasis caused by *C. nivariensis* [ 11 ]. However, Arastehfar et al. (2019) demonstrated that clinical isolates of *C. nivariensis* were sensitive to azoles, polyenes, and echinocandins [ 16 ].

**Figure 2:**
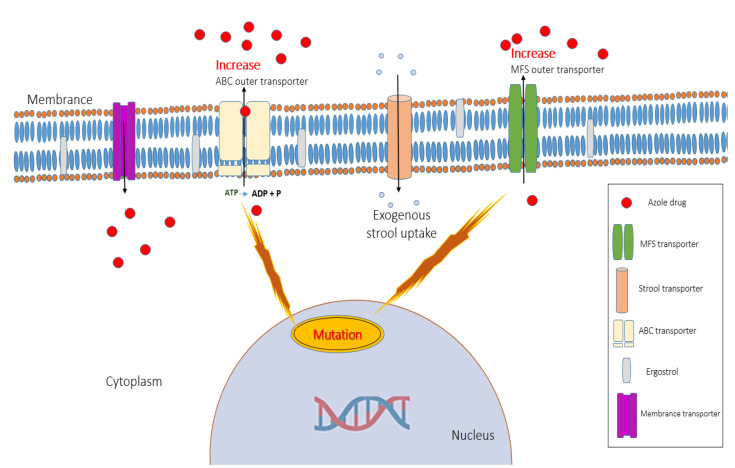
Schematic depiction of the overarching mechanism of azole resistance in *Nakaseomyces glabratus*.

The mechanisms underlying antifungal resistance in the *N. glabratus* complex are currently being extensively investigated. Multiple cases have demonstrated that the *N. glabratus* complex exhibits resistance to azole drugs (Figure 2). These drugs function by inhibition of the 14-α lanosterol demethylase enzyme, which is encoded by the *ERG11* gene. The *ERG11* gene plays a crucial role in ergosterol biosynthesis. Consequently, mutations in this gene result in cross-resistance to both azoles and polyenes [ 17 ].

Various studies have reported that antifungal-resistant *N. glabratus* strains carry mutations in the Pdr1 transcription factor and display increased expression of adenosine triphosphate-binding cassette (ABC)-type efflux pumps, particularly *CDR1* and *CDR2* [ 18 ,  19 ]. These pumps are responsible for the efflux of small molecules from the cell and are regulated by the *TAC1* transcription factor [ 20 ,  21 ].

Mutations in the *ERG11* gene can reduce ergosterol production, thus impairing fungal cell function [ 22 ,  23 ]. As a result, the fungus becomes more vulnerable to antifungal agents and the immune system of the host. Mutations in these genes can lead to dysfunction and subsequent cell death [ 24 ]. The glycolysis pathway genes encode enzymes involved in energy production through sugar metabolism, which is vital for the growth and proliferation of *N. glabratus* [ 25 ].

In addition, the genes associated with the protein synthesis pathway encode enzymes required for protein synthesis, which is essential for cellular functions [ 26 ]. The regulatory genes consist of transcription factor genes, which encode proteins that regulate the expression of other genes, thereby controlling various cellular processes, such as metabolism, growth, and proliferation [ 25 ]. Furthermore, the cell signaling genes encode proteins that facilitate communication between *N. glabratus* and its environment. Cell signaling is crucial for processes, like adhesion to host cells, invasion, and evasion of the immune system [ 27 ].

Notably, the genes associated with this disease include drug resistance genes, allowing *N. glabratus* to withstand antifungal medications.  Expression of resistance genes in *N. glabratus* can be influenced by various factors, including antifungal drug usage, the immune status of the patient, and the specific strain of *N. glabratus* [ 28 ]. Several genes within *N. glabratus* play essential roles in its functioning [ 13 ]. Emergence of azole resistance in clinical isolates of *N. glabratus* has been predominantly associated with the presence of activating mutations in the zinc cluster transcription factor Pdr1 [ 18 ]. These mutations result in differential expression of downstream targets. Rapid occurrence of PDR1 mutations might be attributed to a high frequency of mismatch repair gene *MSH2* mutations, which lead to a hypermutable phenotype [ 5 ,  29 ]. Activating mutations exhibit distinct patterns of downstream effector gene expression, except for increased expression of *CDR1* and *PUP1* [ 30 ].

Several mutations have been identified in the zinc-cluster-containing transcription factor PDR1 in the *N. glabratus* complex, which support the overexpression of transporters *CDR1*, *CDR2*, *SNQ2*, and *PDH1* known for their ability to transport multiple drugs. However, in a study conducted by Lotfali et al.[ 4 ] on azole-resistant *N. glabratus* isolates, no polymorphisms were observed in the complete sequence alignment of the *ERG11* gene. Nevertheless, a mutation in the *FKS1* gene resulting in the substitution of serine with leucine at position 642 (S642L) was observed in the isolates.

It is important to note that even *C. albicans* and *N. glabratus* isolates without mutations in the *ERG11* and *HS1* regions of the *FKS1* gene may still exhibit resistance to azoles and caspofungin. Moreover, no correlation has been observed between the location of a mutation and altered gene expression. Only three genes with direct binding to Pdr1 have been directly implicated in azole resistance: the ABC transporters *CDR1*, *CDR2*, and *SNQ2* [ 31 ].

According to Gygax et al. (2008), a ≥3-fold change in the expression of the *CDR1* gene of *N. glabratus* was found to be associated with resistant isolates [ 10 ]. The present investigation revealed two articles specifically addressing this topic. Notably, in their study, Shi *et al*. (2020) observed that the mRNA expression levels of *ERG11*, *CDR1*, and *CDR2* genes in *C. nivariensis* isolates were higher, compared to those in *N. glabratus* [ 11 ]. This finding suggests that *C. nivariensis* displays a greater degree of resistance. Furthermore, the expression of resistance genes, such as *ERG11*, *CDR1*, and *CDR2* were found to be more pronounced in *C. nivariensis* isolates, compared to *N. glabratus* (Table 2) [ 11 ].

**Table 2:** Resistance genes, involved mechanisms, and results of gene expression in *Candida glabrata* complex isolated from vulvovaginal candidiasis

Yeast	Antifungal agent	Antifungal Resistance		
		Genes and Proteins Involved	Mechanisms Involved	Result
*Nakaseomyces glabratus*	Azoles	Mitochondrial dysfunction associated with the development of mitochondrial DNA-deficient “small” mutants.	Overexpression and activation of ATP-binding cassettes (*CDR1*, *CDR2* [also designated *PDH1*, *SNQ2*, *FAA1*])	• Drugs transported to the outside of the cell
				• Decreased cell surface hydrophobicity during biofilm formation
				• Modification of biological transport pathways of hydrophobic compounds and lipid metabolism
				• Drugs transported to the outside of the cell
*Candida nivariensis*	Azoles	Increased mRNA expression of ERG11, CDR1, and CDR2	Overexpression and activation of CDRs	• Antifungal resistance and increased virulence
				• Increased antifungal resistance
*Candida bracarensis*	NA	NA	NA	NA

* NA: not applicable, ATP: adenosine triphosphate

Recent studies have revealed heightened expression of four major facilitator superfamily (MFS) transporters in clotrimazole-susceptible isolates as opposed to clotrimazole-susceptible clinical isolates [ 32 ]. Perturbing one of these transporters, *TPO3*, resulted in a moderate augmentation of susceptibility to clotrimazole and fluconazole [ 33 ]. These findings imply a subordinate role for MFS transporters in the development of azole resistance [ 22 ].

Since most strains of *N. glabratus* are resistant to azoles, echinocandins are a therapeutic measure that has shown some efficacy. Drugs, such as anidulafungin, caspofungin, and micafungin inhibit the enzyme glucan synthase. These drugs inhibit the formation of β-1, 3-D glucan by binding non-competitively to Fks1p and Fks2p subunits of β-1, 3-glucan synthase. Since β-1, 3-D glucan is an integral part of the structure and function of the fungal cell wall, inhibition of its formation causes high cell wall permeability and, consequently, cell lysis. However, increased resistance to these drugs has been observed in *N. glabratus* complex due to previous exposure to these antifungals. Nevertheless, it seems that since in cases of vulvovaginal candidiasis, low amounts of echinocandin drugs are used, no studies have been conducted in this field.

Nakaseomyces glabratus demonstrates the capacity to thrive with modified plasma membrane sterols, enabling the organism to elude azole treatment. Additionally, *N. glabratus* is capable of assimilating exogenous sterols, regardless of whether the ergosterol biosynthetic pathway is obstructed or under normal conditions in wild-type strains [ 34 ]. Aus1p has been identified as the sterol transporter that is responsible for the tolerance to azoles in the presence of exogenous sterols. Azole resistance in *N. glabratus* has been linked to the emergence of petite mutants, which are characterized by mitochondrial dysfunction and respiratory deficiency [ 35 ].

Understanding the regulation of these genes is crucial for the development of novel antifungal therapies and the prevention and treatment of *N. glabratus*-induced vaginitis.

A limitation of this study was that the available literature in this field was insufficient for conducting a meta-analysis that would enable more robust conclusions. Since the present study aimed to find drug resistance genes only in *N. glabratus* isolates from vulvovaginitis, only three studies could be evaluated.

## Conclusion

Significance of understanding the role of *CDR1* gene in fluconazole resistance cannot be overstated. This gene is central to the development of resistance and will continue to shape the landscape of antifungal therapy and patient management in the years to come. Based on the findings of the texts investigated in this study, the gene expression of azole resistance in *N. glabratus* was associated with vulvovaginitis candidiasis. 

The key insights include a growing trend of resistance in *N. glabratus*, the identification of specific mutations in the *ERG11* and *FKS* genes correlated with resistance, and the clinical implications of the treatment of infections caused by *N. glabratus* complex, such as *C. nivariensis*. It is clear from the compiled evidence that future efforts must be geared toward enhancing our knowledge base, improving diagnostic capabilities, and fostering the development of new antifungal agents. 
